# Characterisation of novel biomass degradation enzymes from the genome of *Cellulomonas fimi*

**DOI:** 10.1016/j.enzmictec.2018.02.004

**Published:** 2018-06

**Authors:** Steven D. Kane, Christopher E. French

**Affiliations:** School of Biological Sciences, University of Edinburgh, Roger Land Building, Edinburgh EH9 3JR, United Kingdom

**Keywords:** *C. fimi*, Arabinofuranosidase, Thermotolerant xylosidase, Multifunctional endoxylanase

## Abstract

Recent analyses of genome sequences belonging to cellulolytic bacteria have revealed many genes potentially coding for cellulosic biomass degradation enzymes. Annotation of these genes however, is based on few biochemically characterised examples. Here we present a simple strategy based on BioBricks for the rapid screening of candidate genes expressed in *Escherichia coli*. As proof of principle we identified over 70 putative biomass degrading genes from bacterium *Cellulomonas fimi*, expressing a subset of these in BioBrick format. Six novel genes showed activity in *E. coli*. Four interesting enzymes were characterised further. α-l-arabinofuranosidase AfsB, β-xylosidases BxyF and BxyH and multi-functional β-cellobiosidase/xylosidase XynF were partially purified to determine their optimum pH, temperature and kinetic parameters. One of these enzymes, BxyH, was unexpectedly found to be highly active at strong alkaline pH and at temperatures as high as 100 °C. This report demonstrates a simple method of quickly screening and characterising putative genes as BioBricks.

## Highlights

• Identified over 90 putative polysaccharide degrading ORFs in *C. fimi* genome.
• Cloned 14 putative cellulolytic ORFs as BioBricks, screened them for activity.
• Partially purified AfsB, BxyF, BxyH and XynF and characterised them further.
• BxyH proved highly temperature and alkaline pH tolerant.
• BioBricks are an easy method for screening genes for specific activities.

## 1 Introduction

Cellulose, the major component of plant cell walls, is the most abundant biopolymer on earth, forming a large part of societal waste [1]. Its structure is made of glucose molecules linked by β-1,4-glycosidic bonds forming long fibres that interact through hydrogen bonds and van der Waal forces to produce microfibrils of crystalline and semi-crystalline cellulose [2]. It's this structure that leads to the majority of the recalcitrance associated with the hydrolysis of cellulose, but the interactions of secondary polysaccharides, termed hemicellulose, also play a major role. For full hydrolysis of cellulose to its composite glucose molecules, the hemicellulose must first be removed. While the structure of the cellulose remains largely the same for all sources, the composition of hemicellulose can vary greatly with different levels of xylan, mannans, arabinose, pectin, ferulic acids and lignin dependent on the plant material [2,3].

In nature the hydrolysis of cellulose requires the concerted effort of a range of enzymes. The accepted model for cellulosic hydrolysis requires the synergistic action of endo β-1,4-glucanases (E.C. 3.2.1.4), exo-β-1,4-glucanases (E.C. 3.2.1.74), cellulose 1,4-β cellobiosidases (E.C. 3.2.1.91) and β-glucosidases (E.C. 3.2.1.21). The hydrolysis of hemicellulose requires a far greater number of enzymes due to the variability of the compositions and structures. The hydrolysis of xylan for example requires endoxylanase and xylosidase activities for the backbone, and then enzymes capable of hydrolysing the branched sugars (arabinofuranosidase, galactosidase, glucosidase) and chemical attachments (feruloyl esterase, acetyl xylan esterase). Different sugar backbones and branching schemes obviously require further enzymes (pectate lyase, chitinase, mannanase, mannosidase, arabinanase, fucosidase, etc.) [3,4]. Enzymes can be just as important for hydrolysis of chemically pre-treated biomass as for hydrolysis of raw cellulosic material [5].

These hemicellulose sugars have been largely considered as contaminants blocking the access of enzymes to the glucose stored as cellulose. Often pentose sugars, these are not readily utilised by many common fermenting species such as *Saccharomyces cerevisiae* [6,7]. The removal of these oligosaccharides is usually performed using physical and chemical pre-treatments, selectively solubilising the sugars before enzymatic treatment of the revealed cellulose [3]. This adds a lot of the cost and complexity to the conversion of biomass to fermentable sugars and often results in the production of fermentation inhibitors as by-products [8].

Consolidated bio-processors (CBPs) are organisms engineered to hydrolyse cellulose *and* ferment the released sugars, and are espoused as a solution to these hindrances [9,10]. Synthetic biology uses modular biological units, which can be combined to create artificial networks to perform specific functions [11]. The hydrolysis of cellulosic biomass by a CBP will entail the combination of genes encoding a wide variety of polysaccharide degrading enzymes. Synergistic enzymes with multiple functions, which operate at similar optimum temperatures and pH values, from sources easy and cheap to produce should reduce the cost associated with lignocellulosic biofuel. Enzymes from the same source organism are most likely to have these qualities.

Cellulolytic, Gram-positive, mesophilic, facultative anaerobic soil bacterium *Cellulomonas fimi* has been studied for over thirty years due to its efficiency for degrading cellulosic material and ease of culturing [12,13]. Prior to the release of the genome by the US DOE [14], there were 14 cellulolytic enzymes known in *C. fimi* (Table 1). Endoglucanases CenA, CenB and xylanase XynB (Cex) were identified from screening of phage lambda libraries in *E. coli* for activity on CMC and avicel [[15], [16], [17]]. XynC and XynD were similarly identified by screening with oat-spelt xylan [18,19], mannanase Man26A and mannosidase Man2A were identified using azo-carob galactomannan and 4-methylumbelliferyl-β-d-mannoside as substrates [20]. *N*-acetyl glucosamidases NagA and NagB were found using 4-methylumbelliferyl β-N-acetyl-d-glucosaminide [21]. Examination of enzymes secreted by *C. fimi* on different substrates and able to bind to sugar led to identification of CenC [22], CenD [23], CbhA [24], CbhB [25] and XynE (Cfx) [26]. Since the release of the genome five β-glycosidases including one bifunctional β-xylosidase [27] and an α-l-arabinofuranosidase [28] have been discovered as a direct result of data mining the genome annotations.

**Table 1:** A selection of putative functions and known gene products required for cellulose degradation found in the *C. fimi* genome.

Putative Enzyme Function	GH Families	Total Novel	Already Known in *C. fimi*
Endo 1,4-β-glucanase	5, 6, 9, 16, 64, 81	12	CenA, CenB, CenC, CenD
1,4-β cellobiohydrolase	6, 48	1	CbhA, CbhB
Endo 1,4-β xylanase	10, 11, 43	3	XynB, XynC, XynD, XynE
Xylan 1,4-β xylosidase	39, 43	5	
α-l-arabinofuranosidase	43, 51, 62	4	AbfCelF(AfsA)
arabinanase	43	2	
β-mannanase/mannosidase	None, 2, 26	2	ManA, ManD
α-amylase	None, 13	9	
Pectate Lyase	None	4	
Chitinase	None, 3, 18, 20	3	NagA, NagB
Acetyl Xylan Esterase	None	3	

The traditional methods used thus far to identify polysaccharide degrading enzymes in *C. fimi* have resulted in a relatively meagre set of enzymes when compared to those predicted for other cellulolytic organism whose genomes are available [[29], [30], [31], [32]]. The further 6 enzymes latterly discovered supports this supposition that there are many useful enzymes still to be discovered in this organism. A simple and rapid means of assessing individual genes is therefore essential. In this paper we report a pilot study for the identification of a range of putative cellulolytic gene products, and the cloning, expression, functional screening and partial characterisation of a subset of these genes using the BioBrick format [11].

## 2 Materials and methods

### 2.1 Chemicals, strains and reagents

All chemicals and reagents were purchased from Sigma-Aldrich unless otherwise stated. *C. fimi* ATCC484 was used as source for all cloned genes. *Escherichia coli* JM109 was used for plasmid construction and gene expression. Primers were synthesised and purchased from Life Technologies.

### 2.2 Gene selection

A keyword search of the uploaded and *C. fimi* genome was carried out to identify putative gene sequences of interest for extraction. DNA was translated into amino acid sequence and searched against databases using BLAST and InterProScan [33] to identify domains and motifs to support the annotation given. From this pool a subset was selected, as proof-of-concept, for cloning and screening based on ease of assay method and their comparability to published and commercially available enzymes.

### 2.3 Cloning and plasmid construction

*C. fimi* genomic DNA was extracted using the Puregene Yeast/Bact. extraction kit (Qiagen) and used as template. PCR cloning of genes was performed using gene specific primers using Kod Xtreme Hot Start DNA Polymerase with GC buffer (Novagen, cat.: 71975). PCR products were purified from solution using Qiagen PCR purification kit (cat.: 28104). Purified product and plasmid Edinbrick1 (pSB1A2-BBa_J33207, a high copy number ampicillin resistance plasmid with *lac* promoter and *lacZα'*, available from the Registry of Standard Biological Parts) were digested by restriction enzymes *Eco*RI-HF and *Spe*I (NEB) following the blueprint of the BioBrick construction method RFC 10 [11] and ligated overnight using T4 DNA ligase (NEB). Ligation mixture was used to transform *E. coli* JM109 using the method of Chung et al. for cell preparation [34]. Transformants were plated on Luria agar containing 100 μg/ml ampicillin, 40 μg/ml X-gal and 90 μg/ml IPTG. White colonies, in which the gene of interest had replaced the P_lac_-*lacZα'*, cassette, were selected for extraction using Qiagen QIAprep Spin Miniprep (cat.: 27104) and plasmid insert confirmed by Sanger sequencing performed by Edinburgh Genomics. Correct constructs were digested using *Eco*RI-HF and *Xba*I, while plasmid pSK1, containing P_lac_-*lacZα*'-RBS BBa_B0034 was digested using *Eco*RI-HF and *Spe*I, mixed and ligated. *E. coli* JM109 was transformed and plated as previously described, with blue colonies, in which the P_lac_-*lacZα'-RBS*, cassette had been incorporated, being selected for plasmid recovery. Sanger sequencing by Edinburgh Genomics was used to confirm the presence of promoter, RBS and gene in correct orientation. *E. coli* containing the correct construct was stored as a glycerol stock at −80 °C.

### 2.4 Expression and activity screening

*E. coli* transformed with the correct final construct were grown overnight in 20 ml LB in the presence of antibiotic and IPTG. Cells were harvested by centrifugation at 7000*g* for 5 min at room temperature, the supernatant discarded and the cells suspended in 0.5 ml PBS (10 mM Na_2_HPO_4_, 1.8 mM KH_2_PO_4_, 137 mM NaCl, 2.7 mM KCl, adjusted to pH7.4 using HCl) and 0.5 ml 50% v/v glycerol and transferred to clean 1.5 ml microcentrifuge tubes pre-chilled on ice. The cells were lysed by sonication through 10 rounds of mixing by inversion, followed by pulsing at 10 kHz for 5 s then incubation on ice for 30 s. The lysate was centrifuged at 14,000 × *g* for 10 min to remove cell debris. The soluble protein fraction was transferred to a fresh microcentrifuge tube, kept on ice or stored at −20 °C when not in use.

#### 2.4.1 Assay procedures

A reaction mixture of 15 μl 50 mM sodium acetate buffer pH 5.0, 5 μl raw cell lysate or purified protein and 5 μl of nitrophenyl substrate was routinely used. 2-nitrophenyl β-d-xylopyranoside (ONPX), 2-nitrophenyl β-d-cellobioside (ONPC), 2-nitrophenyl β-d-galactopyranoside (ONPGal) and 4-nitrophenyl α-l-arabinofuranoside (PNPA) were used to assay for β-xylosidase, β-cellobiosidase, β-galactosidase and α-arabinofuranosidase activities, respectively. The twenty five microlitre reaction mixture was incubated for 1 h at 37 °C then placed on ice to effectively stop the reaction. Immediately before taking the spectrophotometric reading at 405 nm, an equal volume (25 μl) of 1 M Na_2_CO_3_ was added and thoroughly mixed by inversion. All readings were taken using a NanoDrop 2000, using the UV vis setting.

Lysates were assayed for β-glucosidase activity with 4-methylumbelliferyl β-d-glucopyranoside (MUG). Ten microlitres of cell lysate was added to 85 μl of 50 mM sodium acetate, pH 5.0 and 5 μl of MUG (5 mg/ml in H_2_O). The lysates were then incubated at 37 °C for 1 h and the presence of released methylumbelliferol was measured by absorbance at 348 nm using a NanoDrop 2000.

*E. coli* lysate from cells expressing plasmid with no gene insert were used as negative controls for assays, and where possible a *C. fimi* gene of known enzymatic function expressed in the same fashion as the novel genes was used as a positive control. XynB (Cex) was used as positive control for cellobiosidase and xylosidase assays, *Cytophaga hutchinsonii* BglX (CHU2268, C. K. Liu et al., manuscript in preparation) was used as positive control for β-glucosidase assays.

### 2.5 Protein purification

#### 2.5.1 Cell cultures

*E. coli* JM109 expressing the gene of interest were grown in 50 ml volumes of LB containing antibiotic and IPTG overnight. The cells were recovered by centrifugation at 7000 × *g* for 5 min and the pellet suspended in 1 ml of Binding Buffer (BB – 25 mM Tris-HCl, pH 8.0, 25% v/v glycerol). The cells were lysed by sonication as previously described.

#### 2.5.2 Anion exchange chromatography

All purification steps were performed at 4 °C. A 4 ml column of DEAE-Sepharose was washed, equilibrated and regenerated according to recommended operating procedures. The sonicated cell lysate was loaded on the column and washed through with 3 ml of BB. The flow through (FT) and all subsequent fractions were collected. The column was washed three times with 1 column volume (CV) of BB. The protein was eluted in a stepwise fashion, 1 CV of BB containing NaCl in 0.1, 0.2, 0.3, 0.4 and 0.5 M concentrations unless otherwise stated. Fractions were assayed for enzyme activity as previously described. Elution fractions showing activity were pooled and made up to a concentration of 1 M NaCl and used for the next purification step.

#### 2.5.3 Hydrophobic interaction chromatography (HIC)

A 2 ml phenyl-agarose column was washed, equilibrated and regenerated according to recommended operating procedures. The pooled fraction from the previous purification step was loaded onto the column. The column was washed three times with one CV of BB2 (BB plus 1 M NaCl). The proteins were then eluted in a stepwise fashion, with 3*1 ml of BB with 0.5, 0.1, 0.08, 0.06, 0.04, 0.02 and 0 M concentrations of NaCl. Fractions were assayed for enzyme activity as previously described and all fractions were stored at −20 °C.

#### 2.5.4 Cation exchange chromatography

XynF was purified on CM-sepharose after being passed through DEAE-sepharose. The active DEAE fractions were loaded directly to a 1 ml CM-sepharose column. The column was washed 3 times with 1 CV of BB and eluted using 3 times 1 ml BB plus 0.05 M, 0.1 M and 1 M NaCl each. Fractions were assayed for enzyme activity as before.

Purification fractions were analysed by SDS-PAGE, and total protein concentrations estimated by Bradford assay using BSA for the standard curve. After the final purification step if more than one fraction demonstrated enzyme activity, the fraction exhibiting the highest activity:protein ratio was used for characterisation assays.

### 2.6 Enzyme characterisation

#### 2.6.1 pH Optima

Enzymes were assayed as previously described but with increasing pH levels using the following buffers: pH 4.0–5.5, 50 mM sodium acetate buffer, pH 6.0–7.5, 50 mM potassium phosphate buffer, pH 8.0–9.0, 20 mM Tris-HCl buffer and pH 9.5–10.5, 50 mM glycine buffer. All assays were performed at 37 °C.

#### 2.6.2 Temperature optima

The derived optimum pH for the enzyme was used for all temperature experiments. The assays were performed as previously described but at increasing temperatures from 20 °C–70 °C (or higher if needed) in 5 °C steps using a DNA Engine PCR machine (MJ Research) and 0.2 ml thin walled PCR tubes (Axygen).

#### 2.6.3 Effect of supplements

The enzyme specific optimum pH and temperature were used for all subsequent assays. The assays were performed as previously described but with 14 μl of buffer. The remaining 1 μl was one of the following supplements at a final concentration of 1 mM – CaCl_2_, CoCl_2_, CuSO_4_, FeSO_4_, MgCl_2_, MnCl_2_, ZnSO_4_, DTT, EDTA, glucose, cellobiose, xylose or arabinose. FeSO_4_ was made fresh on the day of use as precipitate would form and obfuscate the OD 405 nm readings. All incubations were performed in duplicate with enzyme negative controls.

#### 2.6.4 Enzyme kinetics

Enzymes were diluted to a level where activity was still detectable under normal assay conditions. Enzymes were assayed at their optimum pH and temperature with an increasing level of substrate for 10 min. Precipitation prevented high levels of ONPX (>20 mM) from being used. Enzyme negative controls were used to correct for background noise at high substrate concentrations. All assays were performed in duplicate.

Activity was measured as relative to the maximum activity observed for a reaction minus any enzyme negative background observed. For the purification table (Table 4), standard curves of 2- and 4-nitrophenol at 0, 50, 100, 250, 500, 750 and 1000 μM concentrations at pH 5.0 were used to estimate molar absorption coefficients and fraction activities (1192.6 M cm^−1^ and 9594.4 M cm^−1^, respectively). A unit is defined as the amount of protein required to release 1 μmol of nitrophenol from its bound sugar per minute.

## 3 Results

### 3.1 Putative polysaccharide degrading enzymes

The uploading of the *Cellulomonas fimi* ATC484 genome by the US DOE (NC_015514.1) has allowed the identification of potential ORFs that putatively encode polysaccharide hydrolysing enzymes further to those already identified. The annotation of the uploaded genome was purely automated and was used as a starting point, with further confirmation of identified potential ORF products being based on manual protein domain and BLAST searches, and the putative genes named after the most frequent hit in the top 20. By this method all previously known enzymes were identified along with over 80 other putative polysaccharide degrading enzymes including a further 7 endo-glucanases, 3 putative endo-xylanases, 6 β-xylosidases, and 2 mannanases (Table 1). There appears to be a full complement of hemicellulolytic genes, necessary for the hydrolysis of the highly variable hemicellulose component of plant biomass including 5 α-l-arabinofuranosidases, 2 arabinanases and 11 β-galactosidases, all functions largely unreported in *C. fimi.* Four of the five arabinofuranosidases have no predicted GH family, however they group closely to other characterised GH 51 enzymes when aligned using T-coffee [35] and AfsA (Abfcelf) is now reported as such [28]. These putative genes potentially give *C. fimi* the ability to utilise a wide variety of sugar sources from plants, fungi, insects and animals.

Many of the putative proteins are predicted to have domains for binding to simple sugars, such as the ricin-B-lectin and concanavalin-A like domains. Four carbohydrate binding proteins, with no detected catalytic domain, were also found (Celf_0270, 0403, 1913, 2856). Ten putative genes (Celf_0006, 0651, 0718, 0720, 0735, 1024, 3189, 3746), arabinogalactan-endo-β-galactosidase *abgA* (CelF_3434) and lectin domain containing Celf_3497 were found with LPXTG domains, which is known to anchor to cell surfaces. Also of note are the gene clusters and putative operons which are evident. By looking at the surrounding genes of the putative polysaccharide hydrolases, 30 of these genes are flanked with other genes of related function often less than 100 nucleotides from either the start or the end of the gene. These clusters almost always contain extracellular solute binding proteins and transmembrane transport proteins, presumably for the import of specific released sugars into the cell. These clusters have been observed in other species and may be a vital link in the construction of an efficient CBP [14,32,36,37].

### 3.2 Cloning, expression and activity screening

Table 2 lists the genes cloned as BioBricks from *C. fimi* in this study. These were initially selected as the putative genes coded for industrially useful enzymes, could be easily assayed for and compared to available enzymes. Initially crude *E. coli* cell lysates containing the soluble protein fraction after IPTG induction were used to assay for cellulolytic activity, summarised in Table 3. None of the genes cloned and screened appeared to produce any β-endoglucanase enzymes, with all assayed lysates having levels below that of the negative control, with RBB-CMC as substrate. With RBB-xylan as substrate, XynF proved positive with an absorbance reading well above background and AfsA, AfsB and BxyF showing possible low levels of activity.

**Table 2:** List of *C. fimi* genes cloned and screened in this study.

Gene Name	DOE Annotation	Genome Position	GH Family, Domains	Predicted Function by BLAST	Predicted localisation
*afsA*	α-l-arabinofuranosidase domain containing protein	Celf_3321	probable GH51, α-l-arabinofuranosidase C-terminal	arabinofuranosidase	cytoplasmic
*afsB*	α-l-arabinofuranosidase domain containing protein	Celf_0903	probable GH51, α-l-arabinofuranosidase C-terminal	arabinofuranosidase	cytoplasmic
*afsC*	α-l-arabinofuranosidase domain containing protein	Celf_3249	probable GH51, α-l-arabinofuranosidase C-terminal	arabinofuranosidase	cytoplasmic
*bxyC*	Xylan 1,4-β xylosidase	Celf_3643	GH43, concanavalin A-like glucanase	xylosidase	unknown
*bxyD*	Glycoside Hydrolase Family 43	Celf_1745	GH43, concanavalin A-like glucanase	xylosidase	unknown
*bxyE*	Glycoside Hydrolase Family 43	Celf_0899	GH43, endo-1,5-arabinanse domain	xylosidase	unknown
*bxyF*	Glycoside Hydrolase Family 39	Celf_1744	GH39, alpha-beta hydrolase fold 3	xylosidase	cytoplasmic
*bxyH*	Xylan 1,4-β-xylosidase	Celf_3270	GH39	xylosidase	unknown
*celA*	Cellulase	Celf_0376	GH5, DUF291, Immunoglobulin-like E-set domain, GH5 endoglucanase B, signal peptide	endoglucanase	unknown, not cytoplasmic
*celD*	Glycoside Hydrolase Family 9	Celf_0045	GH9, CBMII, Fibtonectin III, six hairpin glycosidase like domain	endoglucanase	extracellular
*celE*	Glycoside Hydrolase Family 9	Celf_1705	GH9, CenC-like CBM (CBM_4_9), 6 hairoin glycosidase like, galactose binding domain like, immunoglobulin like fold, transmembrane regions, signal peptide	cellobiohydrolase/ glucanase	extracellular
*celF*	Glycoside Hydrolase Family 9	Celf_1481	GH9, 6 hairpin glycosidase like domain	endoglucanase	extracellular
*celN*	Ricin-B-lectin	Celf_2403	GH5, ricin-B-lectin domain	cellulase	extracellular
*xynF*	Endo-1,4-β-xylanase	Celf_0088	GH10, CBMII, TAT signal peptide	xylanase	extracellular
*xynG*	Glycoside Hydrolase Family 10	Celf_1729	GH10	xylanase	extracellular

Domains were identified using Interproscan [33], extracellular localisations by PSortB V3 [52], named based on top hits in blast: afs – arabinofuranosidase, bxy – β-xylosidase, cel – endoglucanase/cellulase, xyn – xylanase.

**Table 3:** Activities of *E. coli* crude cell lysates and after purification (a) against substrates listed (see materials and methods).

Gene (Product putative name)	PNPA	ONPC	ONPX	RBB-CMC	RBB-Xyl	Substrate for Optimal Activity^a^	Temperature (°C), pH optima^a^	V_max_ (U/mg), K_M_ (mM)^a^
Celf_3321 (AfsA)	+	−	+	−	+			
Celf_0903 (AfsB)	+	−	+	−	−	PNPA	45, 6.5	133, 6.8
Celf_3249 (AfsC)	+	−	−	−	−			
Celf_3643 (BxyC)	−	−	−	−	−			
Celf_1745 (BxyD)	−	−	−	−	−			
Celf_0899 (BxyE)	−	−	−	−	−			
Celf_1744 (BxyF)	−	−	+	−	+	ONPX	45, 6.0	326, 1.7
Celf_3270 (BxyH)	−	−	+	−	−	ONPX	80, 9.0	NM
Celf_0376 (CelA)	−	−	−	−	−			
Celf_0045 (CelD)	−	−	−	−	−			
Celf_1705 (CelE)	−	−	−	−	−			
Celf_1481 (CelF)	−	−	−	−	−			
Celf_2403 (CelN)	−	−	−	−	−			
Celf_0088 (XynF)	−	+	+	−	+	ONPC,	60, 6.0	10151,
						ONPX,	50, 4.5	87
						RBB-xyl	40, 5.5	NM
Celf_1729 (XynG)	−	−	−	−	−			

+ = activity detected; − = no detected activity; NM = not measured.

**Fig. 1:**
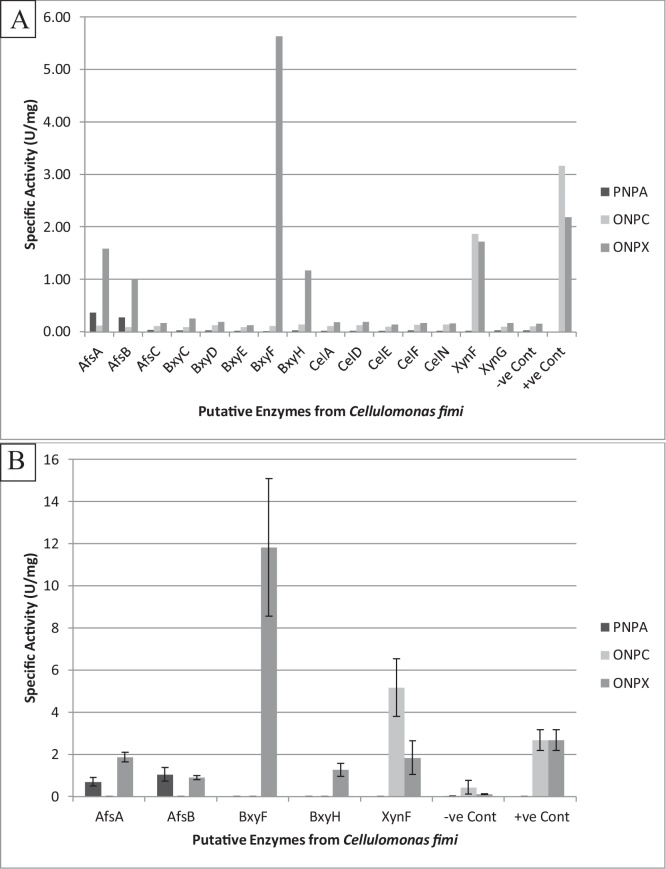
Initial screening data for genes expressed in *E. coli* with A) PNPA, ONPC and ONPX as substrates. Negative (−ve) control for all substrates, plasmid only; Positive (+ve) control for ONPC and ONPX only was Cex. B) Biological repeats of cell lysates displaying positive activity. Mean acitivities of n = 2 independent biological repeats and ± standard error are shown.

Fig. 1A and B shows the specific activities of the cell lysates on specific substrates. AfsA and AfsB showed activity above background levels with PNPA as substrate and activity on ONPX. BxyF proved highly active against ONPX, XynF displayed activity with both ONPC and ONPX as substrates and BxyH displayed activity against ONPX. These initial screens were used to rapidly detect activity on substrates of interest. The other genes showed no detectable activity. ONP-Galactoside was used to assay for β-galactosidase activity as an internal promoter activity control, and was found to be of near identical levels in all cell lysates, indicating that P_lac_ expression was induced to similar levels in all cases. Negative results therefore, may be due to poor translation of a given protein in *E. coli*, or due to lack of activity of the enzyme under the parameters used.

All activities observed in the intial screens were confirmed (Fig. 1B). AfsA and AfsB have both arabinofuranoisdase and xylosidase activities, with AfsA at this temperature and pH being more active with ONPX than PNPA and AfsB being roughly equal. BxyF and BxyH show strong activity with ONPX as substrate but BxyF is far more active in these assay conditions. XynF displays dual activities against ONPC and ONPX with a greater degree of activity shown towards ONPC.

### 3.3 Characterisation of enzyme activities

A subset of the enzymes were purified and characterised further (Table 3, Table 4). AfsA and AfsB showed similar activity patterns, however AfsB displayed higher activity with PNPA as substrate so was selected for purification. Enzymes were purified as described in the methods section, and their main unique activities investigated (arabinofuranosidase for AfsB, xylosidase activity for BxyF and BxyH, and cellobiohydrolase activity for XynF).

**Table 4:** Fold purifications and yields of target enzymes in their first and final purification steps from crude cell lysates of *E. coli*.

Enzyme	Elution fraction	Elution Volume (ml)	Total Protein (mg)	Total Activity (Units*ml)	Specific Activity (Units/mg)	Fold Purification
AfsB	DEAE 0.3 M NaCl	4	2	13.2	26.4	14.8
	HIC 0.1 M NaCl_1	1	0.3	4.4	17.1	9.6
BxyF	DEAE 0.4 M NaCl	6	7.5	47.6	38.1	2.8
	HIC 20 mM NaCl_3	1	0.1	12.6	167.7	12.1
XynF	DEAE FT	4	3	7.3	9.8	3.6
	CM 0.5 M NaCl	1	0.1	10.8	269.7	100.1
BxyH	DEAE 0.5–0.3 M NaCl	8	2.6	1.4	4.4	5.7
	HIC 0.1 M NaCl	2	0.2	2.4	31.7	40.9

#### 3.3.1 Optimum pH

AfsB, BxyF and XynF displayed apparent maximal activity in the neutral range of pH 6–7, 6 and 6, respectively. Outwith those pH values they differ slightly in their tolerances. AfsB activity drops significantly below pH 4.5 or above pH 8, with no activity recorded for pH 4 or pH 9.5 and above, whereas BxyF still retains around 20% of activity at these values. XynF also remains fairly active with nearly 60% activity at pH 4 and 10–20% activity between pH 8.5 and 10.5. BxyH activity was greatly affected by the differing buffers making determining the optimal pH problematic. It was clear however that BxyH activity greatly increased at the alkaline limit of a buffer, and greatest activity was measured at pH 9, which was used for subsequent assays.

#### 3.3.2 Optimum temperature

**Fig. 2:**
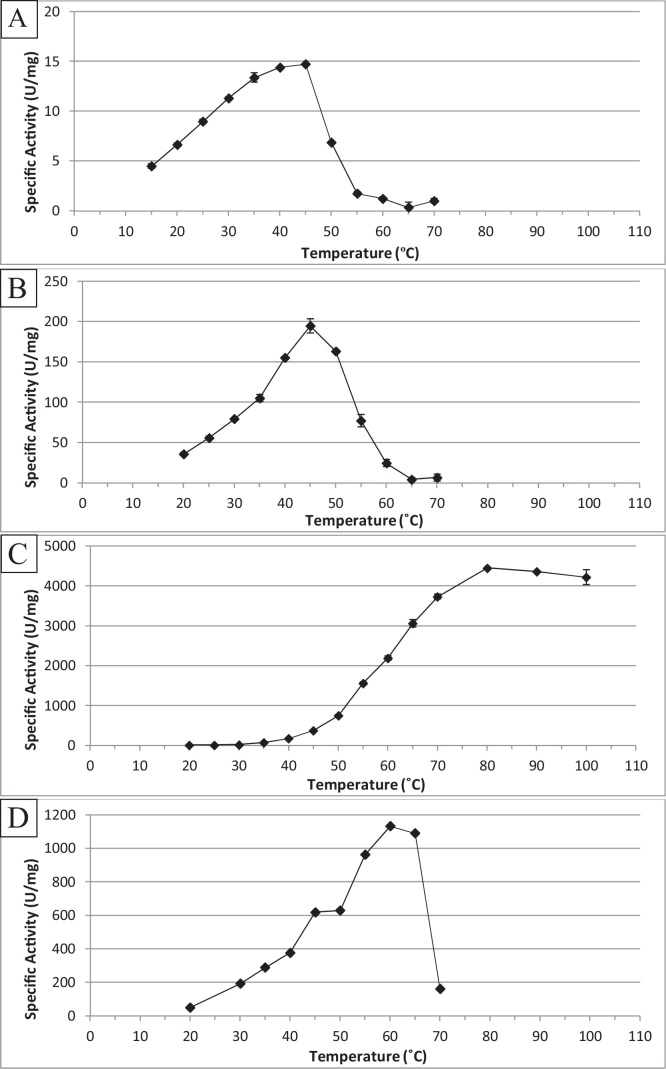
Temperature optima of the purified *C. fimi* enzymes AfsB (A), BxyF (B), BxyH (C), XynF (D). Mean activities (n = 2) of independent biological repeats are shown with ±SE displayed.

Optimum temperatures were determined using the apparent optimum pH environments and are shown in Fig. 2. AfsB (2A) and BxyF (2B) both show 45 °C optima, in the mesophilic range, which might be expected for enzymes originating from *C. fimi*. Their operating temperature range is fairly narrow, with less than 80% of activity detected for AfsB below 30 °C and above 45 °C. BxyF shows less tolerance for colder temperatures, with less than 80% of activity observed below 40 °C, but more tolerance to slightly higher temperatures with 84% activity observed at 50 °C. XynF (2D) with ONPC as substrate displays an optimum temperature at around 60 °C, but with 96% still retained at 65 °C. A steep decline is then observed with as little as 17% activity at 70 °C. BxyH (2C) operates optimally at 80 °C and shows high activity beyond this with 95% of functionality still retained at even 100 °C, far outside what may be expected from a mesophilic organism. Indeed, at 45 °C and 60 °C, the optima for AfsB, BxyF and XynF, the activities of BxyH are only 8% and 50%, respectively.

#### 3.3.3 Effect of metal ions and sugars

**Fig. 3:**
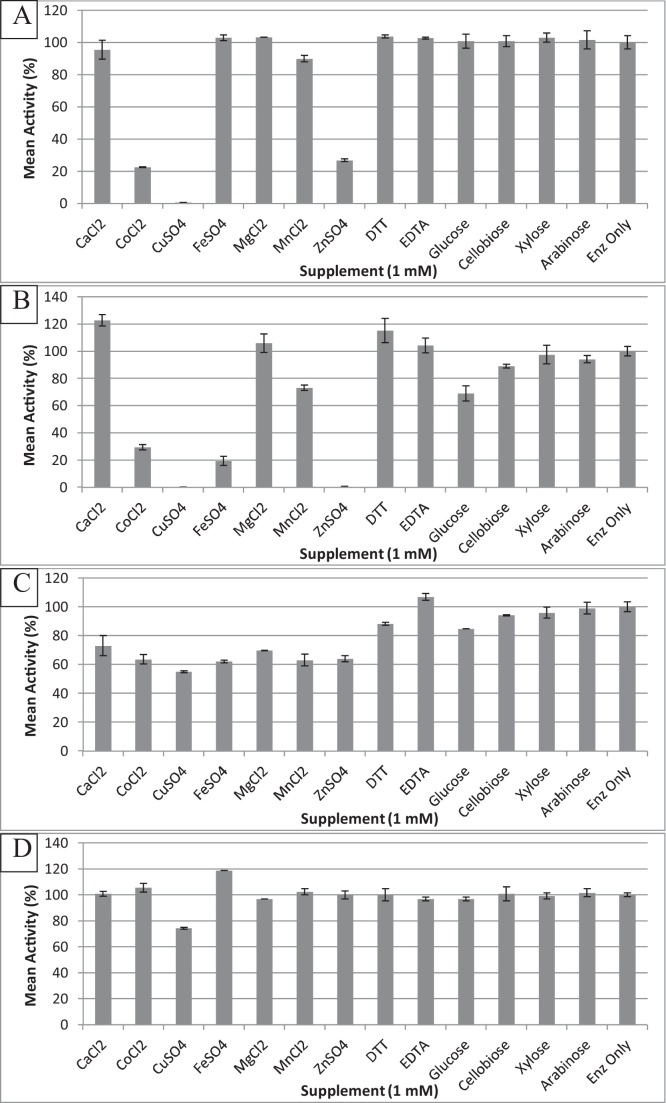
Mean activities (n = 2) of independent biological repeats of enzymes under optimal assay conditions in the presence of varying buffer supplements, all of 1 mM concentration. Enz only is the purified enzyme under assay conditions with no supplement. ±standard error is shown.

As can be seen in Fig. 3 there are seemingly no universally beneficial ions for these enzymes with the most pronounced effects being negative.

At the 5% significance level only Ca^2+^ and Fe^2+^ ions had a positive effect, increasing the activity seen for BxyF by 20% and XynF by 45%, respectively. However, Fe^2+^ had a detrimental effect on BxyF, almost completely inhibiting activity, and enough of an inhibitory effect on BxyH to be significant. The other metal ions tested had either no significant effect or were highly detrimental. Cu^2+^ ions completely disable AfsB and BxyF and significantly reduce the activity of XynF and BxyH. Co^2+^ ions also significantly inhibit the activities of AfsB (reduced to <25% activity), BxyF (<25% activity) and BxyH (<65% activity), with XynF not being significantly affected. Zn^2+^ is another apparent inhibitor of these enzymes with AfsB, BxyF and BxyH all being significantly inhibited, completely in the case of BxyF. BxyH was significantly inhibited by all the metal ions tested except for Ca^2+^.

DTT had no significant ill effects on these enzymes suggesting disulfide bonds are not crucial for enzyme activity or structure. EDTA also had no significant effect suggesting that certain metal ion co-factors are not necessary for enzymatic function. None of the sugars assayed showed any significant effect on enzyme activity. This suggests that at the 1 mM concentration level competitive inhibition of these enzymes is not occurring to a significant degree, but may still inhibit activity at higher concentrations.

#### 3.3.4 Enzyme kinetics

**Fig. 4:**
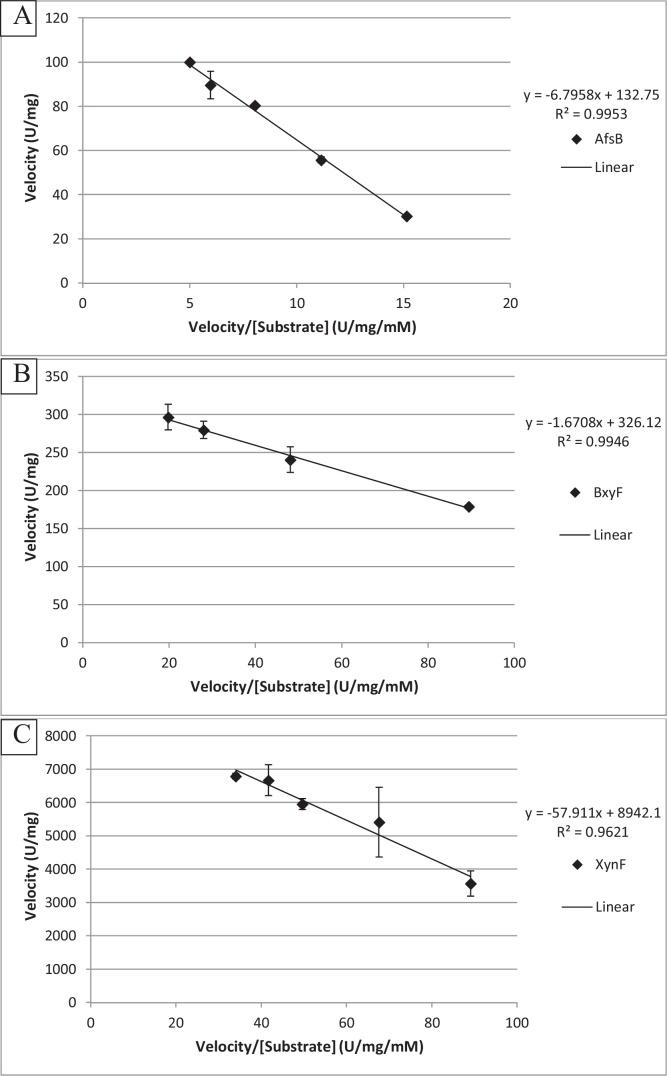
Eadie-Hofstee plots of enzyme velocity (U/mg) as a factor of substrate concentration (mM). A) AfsB, PNPA; B) BxyF, ONPX; C) XynF, ONPC. Error bars of ±SE, n = 2 independent biological repeats.

The solubility of ONPX prevented the kinetics of BxyH from being determined as velocity was still increasing at 20 mM concentration, after which ONPX would precipitate. V_max_ and K_M_ were determined using Eadie-Hofstee plots [38] minus outliers when linearly transformed. AfsB was determined to have a V_max_ of 133 U/mg and K_M_ of 6.8 mM on PNPA (Fig. 4A). For BxyF (ONPX, Fig. 4B), assays at 20 mM and above showed non-linearity, probably due to precipitation of ONPX substrate as was also observed for BxyH. V_max_ was determined as 326 U/mg and K_M_ 1.7 mM using the remaining data points. XynF (ONPC, Fig. 4C) was determined to have a V_max_ of 8942 U/mg and K_M_ of 58 mM.

## 4 Discussion

The main aim of this research was to identify novel enzymes active in *E. coli* from a list of putative genes annotated in the *C. fimi* genome. This paper has shown that the BioBrick format can be used to screen for novel gene functionality in *E. coli*, but could just as easily work for any organism of choice by using a host-specific BioBrick compatible plasmid. The standardised nature of the BioBrick format (i.e. coding sequence only) allows for the automation of both primer design and subsequent PCR in plate form up to 384 genes simultaneously, with the rate-limiting step then becoming plasmid construction and transformation. Once transformants are generated, crude cell lysates are easily obtainable and can be used to screen a wide range of substrates through automation using 96–384 well plates to quickly identify genes of interest, colorimetrically in this paper, with results being widely comparable as the only difference between constructs is the gene ORF.

The modular format of the BioBrick allows genes of interest to be easily swapped into other BioBrick formatted plasmids enabling rapid construction of expression cassettes for a range of host organisms or the construction of multi-part plasmids. The BioBrick therefore has the potential to be a useful tool for the screening of large sets of comparably expressed genes in multiple host environments to increase the number of experimentally verified and correctly annotated enzymes within the databases used for automated annotation, increasing accuracy. With the rapid and increasing number of genomes available a standardised scalable format for functional screening becomes essential.

To date only twenty cellulolytic enzymes have been identified in *C. fimi*. However, similar to the putative gene numbers of other sequenced cellulolytic species [29,31,32,39,40], there was found to be in excess of 90 ORFs coding for putative genes with polysaccharide degrading enzymes as products (Table 1). Further enzymes for degrading simpler sugars include 4 α-galactosidases, 2 α-glucuronidases and 1 α-glucosidase. Almost all have gone unidentified due to the screening methods which generally centred on identifying functionally specific enzymes secreted when grown on a specific and narrow range of simple carbon sources.

Wakarchuk et al. recently published a mass spectrometry secretome analysis of *C. fimi* when grown on CMC or xylan which showed some interesting results [41]. They generated a list of 37 proteins present in the media, excluding any they thought to be intracellular. Six of the previously characterised proteins were identified (CenA, CenD, CbhA, XylC, Cex, and Man26A). They denoted Celf_0088 as being XynE (Cfx) (DQ146941 [26]), but translation of the DNA sequence shows significant differences in amino acid sequence for the first 178 AAs of the protein, following which the sequences are identical. This is the ORF we have named XynF in this paper to avoid confusion. Of the other hits they found AfsA (Celf_3321) was induced with both CMC and xylan and BxyF (Celf_1744) which was induced on xylan only. AfsC (Celf_3249) was also found in low levels for both carbon sources. This indicates that these enzymes are biologically significant, and validates the approach we have taken.

Analysis of the genome has given a more accurate representation of the cellulolytic ability of *C. fimi* but can say nothing of the biochemistry of these proteins, or if they are even real. Genomes are automatically annotated based on other automatically annotated genomes, with little basis in experimental evidence, resulting in a “percolation of errors” [42]. Eleven of our fifteen cloned genes had no characterised enzymes in the top 20 returned hits after a BLAST search. A rapid way to test the most interesting of the annotated genes would greatly enhance the accuracy of annotated genomes and increase the range of potentially industrially useful cellulolytic enzymes.

Of the genes cloned 6 were active against the substrates assayed for (AfsA, AfsB, AfsC, BxyF, XynF and BxyH). This low number could be due to several factors. *C. fimi* is a high G + C (75%) organism, whereas the G + C content of *E. coli* JM109 is much lower (51%). The genes that were assayed as negative may not have been functionally expressed, or expressed at low levels due to the presence of rarer codons truncating the product or causing a bottleneck in translation [43]. The number of active enzymes increased when using an alternative host species *Citrobacter freundii* (supplementary figure SF7). CelD for example was positive for activity on ONPC and ONPX, while BxyC, CelE and XynG became detectably active on ONPX. Similar results were seen by [44], suggesting *C. freundii* may be a superior test system for putative biomass degrading enzymes. The reason for this was not clear nor was it robustly explored further. Another possibility is that the gene products are active against substrates not assayed, which would suggest that the genes may have been incorrectly annotated. It may also be that the temperature and pH conditions were not ideal for these particular enzymes to function, or a mix of all of the above.

Three arabinofuranosidases were identified with AfsA and AfsB being strongly active but AfsC exhibiting very low activity, but still marginally detectable (Table 3). AfsB is a typical bacterial arabinofuranosidase in that it is optimally active at neutral pH 6, as opposed to a more acidic pH as is characteristic of fungal arabinofuranosidases [45]. AfsB's amino acid sequence most closely groups with GH51 by sequence alignment searching. Its optimal values are consistent with those of Yang et al., who report AbfCelf (AfsA) as belonging to GH51, having an optimal temperature of 40 °C, optimal pH of 6.0, V_max_ of 112.4 U/mg and K_M_ of 1.5 mM using PNPA as substrate [28].

The two GH39 β-xylosidases, BxyF and BxyH, are the first of this family of enzymes to be cloned and characterised from *C. fimi*. A bifunctional β-xylosidase/α-l-arabinofuranosidase, active on the corresponding PNP-substrates, has been reported for gene product celf_2726, stating optima of 50 °C and pH of 7.0, but this belongs to GH family 3 [27]. BxyF most closely resembles those that have been previously published, of which there are only 10 GH39 enzymes from bacterial sources characterised according to CAZy [46]. Its optima of pH 6.0 and 45 °C are consistent with the majority of *C. fimi* enzymes identified thus far and a β-xylosidase from *Cellulomonas uda* [47].

Perhaps of most interest is BxyH. Its optimum temperature of 80 °C, with over 90% activity seen as high as 100 °C, would make it of particular interest for industrial applications. These values are higher than those reported for the GH39 xylosidases previously mentioned, and is more akin to temperatures for β-xylosidases from thermophilic bacteria such as *Thermatoga maritima* [48], 90 °C, *Bacillus thermanarcticus* [49], 70 °C and *Geobacillus pallidus* [50], 70 °C to name but a small number. This enzyme has so far been missed by all previous identification methods. Our simple screening technique was able to identify this interesting enzyme. No kinetic data was produced, though it is likely we were not using its preferred substrate, concentrating on a relatively slim list of artificial chemicals for activity screening which could easily be expanded in the future.

The multifunctionality of XynF and being of GH family 10, allows for comparison with the enzymes XynB and XynE from *C. fimi*. XynB and XynE have similar activity ranges to XynF with optimal pH values of 6 and 7, respectively, and optimum temperatures of 40 °C [26,51]. The enzyme kinetics are not comparable as they were determined using different substrates, but the closeness of general characteristics given XynE (Cfx) and XynF maybe the same enzyme further validates this methodology.

## 5 Conclusions

We describe the characterisation of 4 novel enzymes from *C. fimi* cloned as BioBricks: an arabinofuranosidase (AfsB, optima pH 6.5, 45 °C, V_max_ 133 U/mg, K_M_ 6.8 mM), two beta-xylosidases (BxyF, pH 6.0, 45 °C, V_max_ 326 U/mg, K_M_ 1.7 mM; BxyH pH 9.0, 80 °C, undetermined) and an endoxylanase (XynF pH 6.0, 60 °C, V_max_ 8942 U/mg, K_M_ 58 mM). The BioBrick format proves itself a quick method of cloning and testing putative genes with the added benefit of parts being easily assembled into larger networks, necessary for the construction of an organism capable of true consolidated bioprocessing.

## Conflicts of interest

None.

## Appendix A Supplementary data

Primers used, gene clusters, activity and pH data, sequence alignments and *C. freundii* activity data.
