# Supplementary material for: Characterisation of novel biomass degradation enzymes from the genome of Cellulomonas fimi
Source: Enzyme Microb Technol. 2018 Jun;113:9–17. doi: 10.1016/j.enzmictec.2018.02.004 (PMC5892457; doi:10.1016/j.enzmictec.2018.02.004)
Supplement: Supplementary file 1 [file mmc1.docx]

Supplementary Information:

| **Primer Name** | **Sequence (5`-3`)** | **Purpose** |
| --- | --- | --- |
| BioBrick Prefix | tctgaattcccttctagatg... | To lie upstream of coding sequence and include *Eco*RI and *Xba*I restriction sites, to replace native start codon with ATG |
| BioBrick Suffix | tctctgcagctactagtatta,,, | To lie downstream of coding sequence and include *Spe*I and *Pst*I restriction sites, to replace stop codon with TAA |
| AfsAf1 | ...cctcagcgcgccaccgtcc | For cloning the named gene's coding sequence minus start and stop codons |
| AfsAr1 | ,,,gcgggtcagggtcgccgggaggg |  |
| AfsBf1 | ...ttccccgtccgcctcaccc |  |
| AfsBr1 | ,,,ggcgcccaggcggacgacgttcc |  |
| AfsCf1 | ...acccaggcacgactcgtcctc |  |
| AfsCr1 | ,,,gcgggtcagggtcgccgggagg |  |
| BxyCf1 | ...tcgacgacgaccccgcccg |  |
| BxyCr1 | ,,,ggacgcgcgtcccgccgctcc |  |
| BxyDf1 | ...acggaacgcgacccccgg |  |
| BxyDr1 | ,,,tggcctcgtcccttcgaggtgctcg |  |
| BxyEf1 | ...acgctcctggaacccgccgc |  |
| BxyEr1 | ,,,tgccgtccgggtgccgaaggcggtgcgcccgga |  |
| BxyFf1 | ...acgagacgactggtgcccgagc |  |
| BxyFr1 | ,,,cccctgcctcgatgatgtgggtga |  |
| BxyHf1 | ...aaccgcgtcgtcgtccc |  |
| BxyHr1 | ,,,ccctgccggaccgtcgtcgc |  |
| CelDf1 | ...tcacggaacacccaccaccgaggg |  |
| CelDr1 | ,,,ggcgcagggcgacccgttgacgg |  |
| CelEf1 | ...gtcccctcacccgccacgcg |  |
| CelEr1 | ,,,gctgctcgcgcgcgccctccgg |  |
| CelNf1 | ...acgcgacccagaacc |  |
| CelNr1 | ,,,gccgcgcgtccactgctgc |  |
| XynFf1 | ...acgaccccacgtctccacc |  |
| XynFr1 | ,,,ggatgccgcgcacgtgacg |  |
| XynGf1 | ...ccgcaccgccccatcg |  |
| XynGr1 | ,,,ggcgggcaggcgcacctcgac |  |
| pSB1A2insF2 | aataggcgtatcacgacg | Sequencing of pSB1A2 insert site, forward primer |
| pSB1A2insR2 | tttgagtgagctgatacc | Sequencing of pSB1A2 insert site, reverse primer |
| LacZf3 | gggtcgacaggtttcccgactg | Binds at position 215 of BBa_J33207 for sequencing of downstream inserts |
| rbs2f | ccttgaattcgcggccgcttctag  agctcaaggaggtactagtagcg | Construct of RBS BioBrick |
| rbs2r | reverse compliment of rbs2f |  |
| Edinbrick1 | pSB1A2 backbone with BBa_J33207 insert | Insertion of *goi* in pSB1A2 |
| pSB1C3 | pSB1C3 backbone containing BBa_J33207-RBS BioBrick | Insertion of P_lac­_-RBS upstream of *goi* |

Table S1. List of primers and plasmids used in this paper. Prefix sequence found in primers with ...; suffix sequence found in primers with ,,, goi = gene of interest

| **Genomic Region** | **Putative Genes** | **Gene Orientation** |
| --- | --- | --- |
| 578938 – 588738 | *bgaC-f1esb-bpdt-bpdt-liftr-hyp-hyp-abct-tetr* | **🡨🡪🡪🡪🡪🡪🡪🡪🡨** |
| 967746 – 981696 | *liftr-f1esb-bpdt-bpdt-bgaA-bgaB-liftr-abct-imt-imt* | 🡪🡪🡪🡪🡪🡪🡪🡪🡪🡪 |
| 989955 – 997542 | *bxyC-bpdt-bpdt-f1esb-afsB-araB* | 🡨🡨🡨🡨🡪🡪🡪 |
| 1385288 – 1391674 | *liftr-aamD-f1esb-bpdt-bpdt* | 🡪🡪🡪🡪🡪 |
| 1923428 – 1934555 | *bxyF-bxyD-afsD-liftr-f1esb-bpdt-bpdt* | 🡨🡨🡨🡨🡪🡪🡪 |
| 2128016 – 2134275 | *cenD-cbhA-liftr* | 🡨🡨🡨 |
| 2520319 – 2526564 | *bpdt-bpdt-f1esb-aamC-rok* | 🡨🡨🡨🡨🡪 |
| 2549244 – 2560545 | *anat-aamI-bpdt-bpdt-f1esb-liftr-aamB-aamG* | 🡨🡨🡨🡨🡨🡪🡪🡨 |
| 3010411 – 3018856 | *aamA-bpdt-bpdt-f1esb-agaB-rok* | 🡨🡨🡨🡨🡨🡪 |
| 3026808 – 3033512 | *bglG-bpdt-bpdt-f1esb-l1ftr* | 🡨🡨🡨🡨🡪 |
| 3074256 – 3087722 | *rok-f1esb-bpdt-bpdt-hyp-agaA-manA-pbpli-aamH* | 🡨🡪🡪🡪🡪🡨🡪🡪🡪 |
| 3111286 – 3122916 | *bgaK-aglB-bgaE-liftr-afu-bpdt-bpdt-f1esb-bglH-nagC-hyp-hyp-tetr* | 🡨🡨🡨🡪🡨🡨🡨🡨🡪🡨🡨🡨🡨 |
| 3533569 – 3538832 | *afsE-xynE-bxyG* | 🡨🡨🡨 |
| 3629416 – 3637135 | *afsC-bpdt-bpdt-f1esb-luxr* | 🡨🡨🡨🡨🡪 |
| 3649422 – 3667427 | *liftr-mr-aglA-gt36-xynH-bgaF-f1esb-bpdt-bpdt-bglF-liftr-axeB* | 🡨🡪🡪🡨🡨🡨🡨🡨🡨🡨🡪🡪 |
| 3879031 – 3883795 | *bglA-mfs1-l1ftr* | 🡨🡨🡪 |
| 4061326 – 4076123 | *bgaL-rok-bgaG-bpdt-bpdt-f1esb-abct-rbl* | 🡪🡨🡨🡨🡨🡨🡪🡪 |

Table S2**.** Putative polysaccharide-degrading gene clusters

Polysaccharide degrading genes: *aam** – α-amylase; *afs** – Arabinofuranosidase; *afu* – α-fucosidase; *aga** – α-galactosidase; *ara** – Arabinanase; *axe** - Acetyl xylan esterase; *bga** – β-galactosidase; *bgl** – β-glucosidase; *bxy** – β-xylosidase; *xyn** - Xylanase

Transcriptional regulators: *liftr* – LacI family transcriptional regulator; *luxr* – LuxR family; *pbpli* – Periplasmic binding protein/LacI transcriptional regulator; *rok* – ROK family protein; *tetr* – Transcriptional regulator TetR

Transport: *abct* – ABC transporter-like; *bpdt* – binding-protein-dependent transport systems inner membrane component; *f1esb* – family 1 extracellular solute binding protein; *imt* – inner-membrane translocator

Other: *anat* – aminoglycoside N(3`)-acetyltransferase; *gt36* – Glycosyl transferase family 36; *hyp* – Hypothetical protein; *mr* – Mandelate racemase/muconate; *rbl* – Ricin-B-lectin

**Endo-β-glucanasae and endo-β-xylanase activity assays:**

Endoglucanase and endoxylanase activity was assayed for using remazol brilliant blue (RBB) labelled carboxymethyl cellulose (CMC) and beech-wood xylan, respectively. The RBB-CMC was purchased from Megazyme (cat.: S-ACMCL). RBB-xylan was produced as follows, as described by MA Speer, K Demarco and E McCann at <http://www.openwetware.org/index.php?title=Xylanase_Protocols&oldid=578448>

1. Five hundred milligrams of Birchwood xylan (sigma) and 500 mg of remazol brilliant blue were added to 12 ml of deionised sterile water and mixed thoroughly.

2. While being stirred four millilitres of (135 mg in 4 ml) sodium acetate (NaAc) solution was added slowly over a 5 minute period.

3. Four millilitres of sodium hydroxide (NaOH, 300 mg in 4 ml) was added and the solutions mixed for 90 minutes at room temperature.

4. Two volumes of 96% (v/v) ethanol were added to precipitate the RBB-Xylan.

5. This was then filtered with a vacuum flask and Whatman filter paper.

6. The solid material was washed with wash solution (132 ml EtOH, 66 ml H2O, 270 mg NaAc) until the flow through ran clear.

7. The precipitate was then washed with 20 ml 75% (v/v) ethanol, and 4 ml of 100% acetone.

8. The RBB-Xylan was left to air dry overnight. The dry weight was measured, autoclaved and dissolved in sterile de-ionised water to a final concentration of 5% (w/v) and stored at 4ºC.

Reaction volumes of one hundred microlitres were used for the assays containing 0.2% (w/v) RBB-CMC or RBB-xylan, 20 μl cell lysate, and then made up to 100 μl with 50 mM sodium acetate buffer, pH 5.0. The mixture was thoroughly mixed by pipetting and incubated for 2 hours at 37ºC. The reaction was stopped with the addition of three volumes of 100% ethanol and thoroughly mixed. After a one minute incubation at room temperature the mixture was centrifuged at 14,000xg for 10 minutes to pellet the precipitated polysaccharides. The soluble fraction was spectrophotometrically measured using a NanoDrop 2000 at 590 nm.

SF1. Activities of cloned gene products on RBB-dyed soluble cellulose (CMC, blue) or beech-wood xylan (Xyl, red). CenA, CMC positivie (+ve) control; plasmid only, negative (-ve) control; Cex, Xyl positive control

SF2. Enzymatic activity of purified *C. fimi* enzymes AfsB at differeing pH values. Shown as percentages of the maximal activity recorded. Standard error is shown as error bars, n=2. Floating numbers are specific activity of the enzyme at each point, the red line indicates μmol per min of nitrophenol released in enzyme negative assays (2^nd^ y axis)

SF3. Enzymatic activity of purified *C. fimi* enzymes BxyF at differeing pH values. Shown as percentages of the maximal activity recorded. Standard error is shown as error bars, n=2. Floating numbers are specific activity of the enzyme at each point, the red line indicates μmol per min of nitrophenol released in enzyme negative assays (2^nd^ y axis)

SF4. Enzymatic activity of purified *C. fimi* enzymes BxyH at differeing pH values. Shown as percentages of the maximal activity recorded. Standard error is shown as error bars, n=2. Floating numbers are specific activity of the enzyme at each point, the red line indicates μmol per min of nitrophenol released in enzyme negative assays (2^nd^ y axis)

SF5. Enzymatic activity of purified *C. fimi* enzymes XynF at differeing pH values. Shown as percentages of the maximal activity recorded. Standard error is shown as error bars, n=2. Floating numbers are specific activity of the enzyme at each point, the red line indicates μmol per min of nitrophenol released in enzyme negative assays (2^nd^ y axis)

0088 M-----TTPRLHRRGRLA--AAVGGLAAATLAVALAVPAAAAGSTLQAAA

3156 MHTKLHATPRHGWRPRAAALAATAGLVLTTFA-AISTPAQAA-STLGASA

Cfx MHTKLHATPRHGWRPRAAALAATAGLVLTTFA-AISTPAQAA-STLGASA

* :*** * * * **..**. :*:* *::.** ** *** *:*

0088 AESGRYFGTAIAASRLSDGTYTGIANREFNMITAENEMKMDATEPNRGQF

3156 AEKGRYYGTAIAAGRMGDSTYMTIANREFNMITAENEMKMDATEPSQGRF

Cfx AEKGRYYGTAIAAGRMGDSTYMTIANREFNMITAENEMKMDATEPSQGRF

**.***:******.*:.*.** **********************.:*:*

0088 SYSNGDRIVNWARQNGKQVRGHALAWHSQQPGWMQNLSGTDLRNAMLNHV

3156 TFTNGDRIVNWALSNGKRVRGHTLAWHAQQPGWMQSMSGSALRNALINHV

Cfx TFTNGDRIVNWALSNGKRVRGHTLAWHAQQPGWMQSMSGSALRNALINHV

:::********* .***:****:****:*******.:**: ****::***

0088 TQVATYYRGKIYAWDVVNEAYADGSSGARRDSNLQRTGNDWIEAAFRAAR

3156 TQVASYYRGKVYAWDVVNEAFADDGRGSRRDSNLQRTGNDWIEAAFRAAR

Cfx TQVASYYRGKVYAWDVVNEAFADDGRGSRRDSNLQRTGNDWIEAAFRAAR

****:*****:*********:**.. *:**********************

0088 AADPNAKLCYNDYNTDNWSHAKTQGVYNMVKDFKARGVPIDCVGFQAHFN

3156 AADPGAKLCYNDYNTDGI-NAKSTAVYNMVRDFRSRGVPIDCVGFQAHL-

Cfx AADPNAKLCYNDYNTDNWSHAKTQGVYNMVKDFKARGVPIDCVGFQAHFN

****.***********. :**: .*****:**::*************:

0088 SGNPVPSNYHTTLQNFADLGVDVQITELDIEGSGSSQAQQYQGVVQACLA

3156 -GTSLPSDFQANLQRFSDLGVEVQLTELDIQ-QGSNQANMYAQVTNACLA

Cfx SGNPVPSNYHTTLQNFADLGVDVQITELDIEGSGSSQAQQYQGVVQACLA

*..:**::::.**.*:****:**:*****: .**.**: * *.:****

0088 VSRCTGITVWGVRDTDSWRASGTPLLFDGSGNKKAAYTSVLNALNAGGTT

3156 VARCTGITVWGVRDSDSWRTGANPLLFDASGNKKPAYTSVLNALNAGGSN

Cfx VSRCTGITVWGVRDTDSWRASGTPLLFDGSGNKKAAYTSVLNALNAGGTT

*:************:****:...*****.*****.*************:.

0088 TPTPNPTNPTPTPTPTNPGGGSPSCTA-----------------------

3156 ------------------GGGSSSIEAGAWYVLVNRNSGKALDVYNLATN

Cfx TPTPNPTNPTPTPTPTNPGGGSPSCTA-----------------------

****.* *

0088 ----------TYSEGQKWGDRFNGTVTIRATTNISSWQSTVTVRSPQK--

3156 DGARITQWTRNDGSQQQWQFQLNSDGYYEVKSRLSGKNLDVSGKSTADGG

Cfx ----------TYSEGQKWGDRFNGTVTIRATTNISSWQSTVTVRSPQK--

. .. *:* ::*. ...:.:*. : *: :*. .

0088 IIATWNGS----PTWD----SSGNVMTMRPSGSGALAAGQSTSFGFTVQH

3156 AIVQWTDNDGANQQWRATVTNGYATLISRLSGKALEVQGASTADGANIVQ

Cfx IIATWNGS----PTWD----SSGNVMTMRPSGSGALAAGQSTSFGFTVQH

*. *... * .. .: * **.. . * **: * .: :

0088 NG-----NWTWPSVTCAAS

3156 YTSWNGANQQWQLV--RVG

Cfx NG-----NWTWPSVTCAAS

* * * ..

SF6. Sequence alignment using T-Coffee (Notredame et al., 2000) of Celf_0088 and Celf_3156 translated amino acid sequences against the published sequence of Hekmat et al, 2005 for Cfx (Hekmat et al., 2005)

**B**

SF7. Activity screenings of cloned genes with *Citrobacter freundii* as host with A) PNPA, ONPC, ONPX and B) RBB dyed CMC and xylose. Positive controls for A) were taken from *E. coli* screen data

Hekmat, O., Kim, Y.W., Williams, S.J., He, S.M., Withers, S.G., 2005. Active-site peptide "fingerprinting" of glycosidases in complex mixtures by mass spectrometry - Discovery of a novel retaining beta-1,4-glycanase in *Cellulomonas fimi*. *Journal of Biological Chemistry*, 280, 35126-35135.

Notredame, C., Higgins, D.G., Heringa, J., 2000. T-Coffee: A novel method for fast and accurate multiple sequence alignment. *Journal of Molecular Biology*, 302, 205-217.
